# Should you be worried?

**DOI:** 10.1007/s12471-014-0527-y

**Published:** 2014-02-13

**Authors:** J. Elias, A. R. Willems, A. A. M. Wilde

**Affiliations:** 1 Department of Cardiology, Sint Lucas Andreas Hospital, Jan Tooropstraat 164, 1061 AE Amsterdam, the Netherlands; 2 Academical Medical centre Amsterdam, Meibergdreef 9, Postbus 22660, 1100 DD Amsterdam, the Netherlands

A 23-year-old man was referred to the emergency room with complaints of dyspnoea and dizziness followed by rapid palpitations. He had no relevant medical history and was not using any medication. The family history was negative for arrhythmias and sudden death. Physical examination revealed a tachycardia of 160 beats/min with a normal blood pressure; also, a frog sign was visible. Initial laboratory tests and echocardiography did not demonstrate any abnormalities. After administration of adenosine 9 mg intravenously, the tachycardia was terminated and there was conversion to sinus rhythm.

**Fig. 1:**
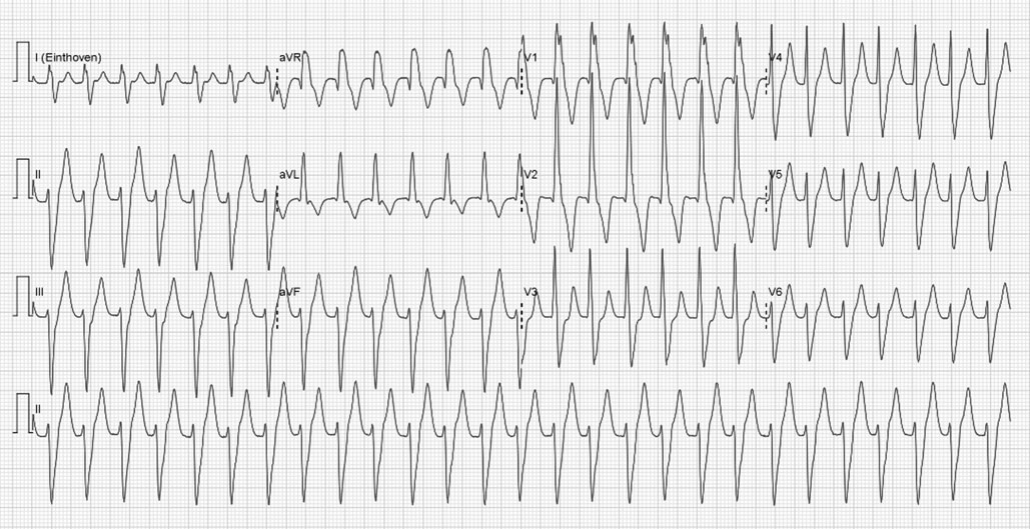
Twelve-lead ECG at presentation

His electrocardiogram is shown in Fig. 1. What is your diagnosis?

Answer

You will find the answer elsewhere in this issue.

